# Letter from Tennessee

**Published:** 1872-07-15

**Authors:** F. K. Bailey

**Affiliations:** Knoxville, Tenn.


### Domestic Correspondence.

### LETTER FROM TENNESSEE.

KNOXVILLE, Tenn., May 13, 1872.

*Editor of Examiner:* Our winter was unusually protracted; and, consequently, many who were unprotected from cold, suffered. In January, catarrhal and bronchial affections were almost universal, affecting all classes and conditions, more or less. As usual, however, the poorer people suffered most, and particularly the blacks. Bronchopneumonia was quite prevalent, and, in many cases, lingered, finally proving fatal. Whooping cough has been unusually scarce in consequence of complications usually seen in spring. There has been more asthenia attending disease for the last six months; and tonics are generally called for.

Menstrual irregularities have been very common. Both amenorrhœa and menorrhagia are daily met with; and uterine diseases still continue frequent. In menorrhagic and leucorrhœal conditions of the womb, I have lately used ergot with great benefit.

In conjunction with bromide and iodide of potassium, according to individual indications, it has proved unfailing in giving relief to the usual dragging pain in the lumbar region, and in checking inordinate flow, both of blood and the usual catarrhal secretions· Coupled with uterine "weaknesses," is commonly found constipation from torpor of the large intestines; and the combination of aloes with other agents has generally been attended with satisfactory results. Ext. belladonna, in doses of from one-fourth to one-half grain, I have found useful in the constipation of females as well as males.

The Knox County Medical Society holds meetings on each alternate Friday night; and, with a few exceptions, a paper has been read and its contents discussed at each meeting.

The rapid disintegration of the lungs in colored girls at puberty, to which I have at former times alluded, still continues common. In looking over the medical literature of the Southern States, I find frequent allusions to this affection, which is known as African consumption. It is generally accompanied with more or less mesenteric disease, in the form of enlargement of the glands. The mesenteric complication is very common in cases of broncho-pneumonia of young children among the blacks. The mortality among the colored population is fearful; and I am inclined to think that at least one-half the children die before they are two years old. Those having a mixture of Anglo-Saxon blood, are universally less enduring; and the bleaching process appears destructive to the race. There is much less sickness among the unmixed, of all ages; but we see quite a proportion of the children born during the last ten years, with white blood in their veins. I may be intruding upon the province of those particularly interested in social science, to enlarge farther upon this point.

Having been engaged, of late, in compiling facts relative to the early surgery of East Tennessee, I find some very interesting items. I met, this morning, with Dr. J. G. M. Ramsey, the historian of Tennessee, who commenced practice in this town in 1820. In mentioning the name of an old pioneer physician of this region (Dr. Wright), he alluded to his plan of arresting arterial hæmorrhage in a case of incised wound of the foot, in which he poured a stream of boiling water from a tea-kettle upon the bleeding vessel. The blood was immediately stopped. He also mentions a traditionary account of the manner of treatment employed by an uneducated practitioner during the war of the revolution, in the case of a gunshot wound extending into the abdominal cavity. By reason of hæmorrhage, the cavity was filled with coagulum before reaching the man. He took equal quantities of warm milk and water, and poured it through a funnel into the cavity, and directed that the patient be rolled gently from side to side, which had the effect to dissolve the clot, and render it practicable to remove it. Then, placing the man upon his face, the whole was allowed to run out; and thus, by a diluting and rinsing process, he saved the life of the sufferer. The Doctor was unlearned in surgical authority, and only followed the dictates of common sense in the case.

For about a half century after the first settlement of this region, there was but little intercourse with the Atlantic cities; and medicine, as well as everything else, was primitive and simple.

Among the earlier physicians that settled in East Tennessee, was Dr. Joseph C. Strong, who graduated at New Haven, Conn., engaged as Surgeon in the United States Navy, till his health failed, and he finally made his way hither, locating in Knoxville about the year 1805. Dr. Strong built almost the first brick house, which now stands, after about fifty years, in a good state of preservation. An old lady died here about a year ago, who, for more than forty years, took the lead in midwifery. She was employed extensively among the slave population, as well as in the white families. In ordinary cases she was employed, and physicians called in if any complication occurred.

Since the war, midwives are less employed than formerly; but still many women, as in all places, hold themselves in readiness to attend their suffering sisters in child-bed.

Puerperal diseases are not common here; and it is very seldom that a woman dies during or after confinement.

There are not far from twenty regular physicians in the city, five homœopaths, and some low quacks, who cannot be well classified. There is one lady physician claiming to be a graduate from a woman's Medical College in Philadelphia.

Many of the patrons of the homœopaths are people from the North, who either employed them before coming, or call upon them here through fear of the "strong medicine" which they are told Southern physicians give.

It is amusing to hear the various reasons given by way of apology for swallowing the pretended "system" as well as pellets. Some will say they have no confidence at all in the doctrine or practice, but at once, like our father Adam, say, "the woman beguiled me." Thus they attempt to wipe from their cheeks the blush of shame which mantles them, while pleading guilty to their want of decision.

